# Diabetes Resolution and Work Absenteeism After Gastric Bypass: a 6-Year Study

**DOI:** 10.1007/s11695-017-2642-5

**Published:** 2017-03-14

**Authors:** E. Jönsson, P. Ornstein, H. Goine, J. L. Hedenbro

**Affiliations:** 1 Swedish Social Insurance Agency, Stockholm, Sweden; 2 Department of Statistics, Uppsala University, Uppsala, Sweden; 3 Department of Medical Sciences, Occupational and Environmental Medicine, Uppsala University, Uppsala, Sweden; 4 Department of Surgery, Clinical Sciences, Lund University, Nicolovius 7, SE 224 65 Lund, Sweden

**Keywords:** Absenteeism, Benefits, Complications, Cost, Gastric bypass, Obesity, Sickness

## Abstract

**Background:**

Obesity-related diseases cause costs to society. We studied the cost of work absenteeism before and after gastric bypass and the effects of postoperative diabetes resolution.

**Patients and Methods:**

Data were obtained from the Scandinavian Obesity Surgery Registry (SOReg) (national coverage >98%) and cross-matched with data from the Social insurance Agency (coverage 100%) for the period ±3 years from operation. In 2010, a total of 7454 bariatric surgeries were performed; the study group is 4971 unique individuals with an annual income of >10,750 Euros and complete data sets. A sex-, age-, and income-matched reference population was identified for comparison.

**Results:**

Patients with obesity had preoperatively a 3.5-fold higher absenteeism. During follow-up (FU), the ratio relative to the reference population remained constant. An increase of 12–14 net absenteeism days was observed in the first 3 months after surgery. Female sex (OR 1.5, CI 1.13–1.8), preoperative anti-depressant use (OR 1.5, CI 1.3–1.9), low income (OR 1.4, CI 1.2–1.8), and a history of sick leave (OR 1.004, CI 1.003–1.004) were associated with increased absenteeism during FU. Diabetes resolution did not decrease absenteeism from preoperative values.

**Conclusions:**

Patients with obesity have higher preoperative absenteeism than the reference population. Operation caused an increase the first 90 days after surgery of 12–13 days. There were no relative increases in absenteeism in the next 3 years; patients did not deviate from preoperative patterns but followed the trend of the reference population. Preoperative diabetes did not elevate that level during FU; diabetes resolution did not lower absenteeism.

## Background

The beneficial effects to patients of gastric bypass surgery (RYGB) are well documented for quality of life [1], for resolution of comorbidities [2, 3], for some cancer forms [4], and also for mortality [5], particularly for diabetics [6]. Also, the direct and indirect costs of the operation per quality-adjusted life year (QUALY) have been estimated to be low and after >15 years lead to reduced societal cost [7]. But some studies suggest that RYGB may also induce problems and thus costs to health care providers. Recently, emphasis has been given to the problem of internal herniation, where between 2 and 10% of patients have been reported to undergo a second operation [8, 9]. Also, an increased risk of hospitalization for alcohol-related disease has been suggested for gastric bypass patients [10]. When considering costs, absenteeism due to such complications must then be weighed against the beneficial effect on comorbidities of the surgery. By using crude absenteeism data, regardless of cause, the aim of the present study was to examine whether surgery gave increased societal absenteeism costs or whether surgery by causing the disappearance of comorbidity led to a reduction in absenteeism.

## Patients and Methods

### Registries

Sweden has national registries using unique individual-specific identification numbers. The Scandinavian Obesity Surgery Registry (SOReg) is fully comprehensive and covers all operations, regardless of hospital and financing [11]. It covers >98 of all bariatric surgery performed and its database now comprises over 53,000 operations. Pre- and postoperative comorbidities, operative and postoperative complications, and basic data are registered, and the database has recently been validated [12]. The operational definitions in SOReg are such that a comorbidity exists if it renders a patient to be on continuous physician-prescribed medication. For type 2 diabetes (T2D), also non-medicated patients with a baseline HbA1c >48 mmol/mol are included (corresponding to ≥6.5%). The variable is thus dichotomous. Resolution of T2D is also dichotomous and defined in SOReg as the combination of a normal HbA1c and no ongoing medication. Information on absenteeism and income was made available through the Swedish Social Insurance Agency’s administrative registries, covering 100% of the population. Individuals with a pension-generating income are insured through this agency, which reimburses work absenteeism from the third week onwards, with the need for medical certification [13].

### Patients

All patients operated in Sweden with a primary RYGB in 2010 were identified from the SOReg. This year was chosen to permit a 3-year follow-up (FU) period and allowing for up to 1 year delay in database management.

**Fig. 1:**
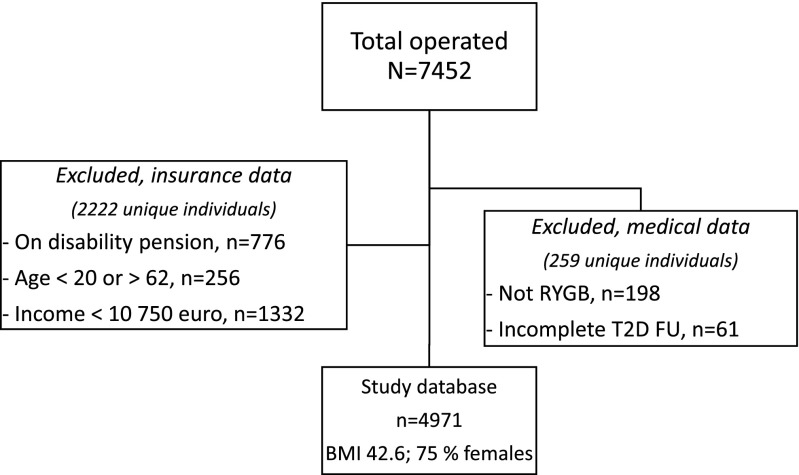
Patient cohort for analyses. *T2D FU* type 2 diabetes follow-up

This cohort of patients was then cross-matched for each individual with the database of the Swedish Social Insurance Agency. Patients having had only a revisional operation in 2010 were excluded. Absenteeism was measured using the amounts of reimbursement from the Social Insurance Agency, for insured individuals. The study group was thus restricted to patients with access to social insurance, operationalized through the following characteristics in 2010: age in the range 20–62 years, no disability pension, and a declared income of >10,750 Euros. An overview of the cohort of operated patients is given in Fig. 1.

### Reference Population

The reference population was created from the Swedish Social Insurance Agency database through age, sex, and income matching. It is based on individuals living in Sweden during 2010 without disability pension and with a declared income from employment of >10,750 Euros. The match was made by identifying individuals with the same age and sex and choosing the closest ten individuals in terms of income. Any individual with the exact same age, sex, and income was excluded from the reference population so that no one from the operated population would be included in the reference population.

### Statistics

The study database has been analyzed at the Swedish Social insurance Agency, which is responsible for official statistics regarding absenteeism reimbursements. All analyses were conducted using the SAS Enterprise Guide version 7.1. First, we measured the total number of days with any sickness benefit, for each 3-month period starting 3 years before the date of surgery until 3 years postoperatively, i.e., a total of 24 data points for each included operated individual and for corresponding individuals in the reference group. To quantify societal cost, the number of days with benefits was multiplied with the actual percentage of absenteeism. Two days with 50% absenteeism thus equals one net day; results are expressed as such net days. This method measures, rather than calculates, societal costs and is used by the Swedish National Board of Health and Welfare [14].

The relative change, per 3-month period, was examined in order to account for different levels of sick leave in the study groups and the reference population. The reason for this is that due to the large difference in preoperative absenteeism between the patient and reference population, a small general percentage change in absenteeism would generate a large shift in the absolute difference between the groups. Next, heterogeneity within the patient group was analyzed. Patient data were stratified for income levels and for age and finally according to the existence of any comorbidity, specifically of type 2 diabetes (T2D). Absenteeism data for patients with diabetes was further sub-classified for resolution or no resolution postoperatively. In addition, simple logistic regression was performed for analyzing the risk of sick leave from 90 days to 3 years postoperatively.

Baseline data from both patients and the reference population are presented in Table 1. The level for statistical significance was set at *p* < 0.05.

**Table 1:** Demographics of operated patient cohorts, separated for those meeting or not meeting the criteria for inclusion in the study, and data for the reference population. Reasons for exclusion from study are given in Fig. 1. Patient data are from time of operation; data for reference population from time of data acquisition (mean (SE))

	Operated patients, not included in the study due to insurance ineligibility	Operated patients, included in the study	Reference population
Number of subjects	2222	4971	49,710
Age			
Female	41.9 (0.31)	40.4 (0.15)	40.5 (0.05)
Male	44.4 (0.66)	42.8 (0.27)	42.8 (0.10)
BMI			
Female	43.0 (0.14)	42.3 (0.09)	n.a.
Male	44.0 (0.30)	43.7 (0.17)	n.a.
Waist circumference (cm)			
Female	125.1 (0.33)	123.8 (0.23)	n.a.
Male	138.4 (0.70)	138.0 (0.40)	n.a.
Declared annual income, Euros			
Female	10,318 (213.0)	28,394 (187.4)	28,393 (59.3)
Male	11,193 (508.2)	35,637 (514.8)	35,637 (162.7)
Pre-op prevalence of T2D	20.5% (0.8)	13.2% (0.5)	n.a.
Resolution of T2D at 1 year FU	57.8% (2.5)	68.2% (1.9)	n.a.

*n*.*a*. not available

## Results

A total of 7454 patients were identified from SOReg coded for having undergone a bariatric procedure in 2010 at any Swedish surgical department for a total population of 9.2 million. The inclusion criteria restricted the patient database to 7195 (97%) unique individuals with complete data sets and further to 4971 (67%) individuals who were eligible for sickness insurance (Fig. 1). Basal morphometric data for excluded and included patients as well as for the reference population are given in Table 1. Matching for age, sex, and income achieved balance for these characteristics between included patients and the reference population. Among the operated patients, excluded patients had a higher prevalence of T2D (*p* < 0.05) and also a lower resolution rate (*p* < 0.001) compared with the included patients. However, as subjects in this group lack access to social insurance, their absenteeism is non-distinguishable and its costs neither measurable nor a cost to society.

**Fig. 2:**
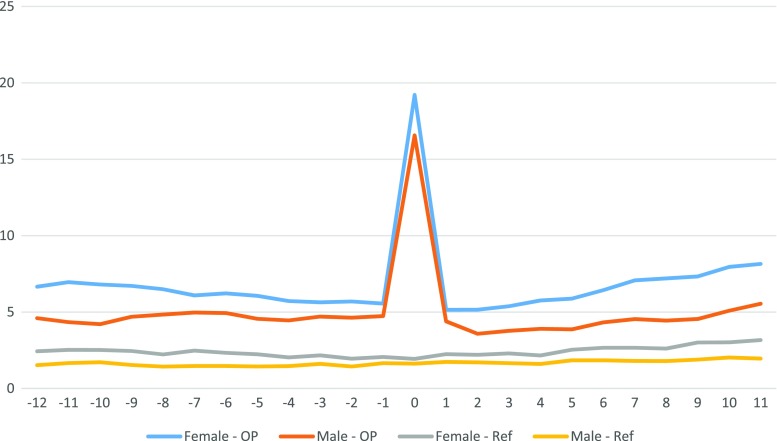
Number of net reimbursed absenteeism days per 3-month period, −3 to +3 years from operation, separated for gender and group

**Fig. 3:**
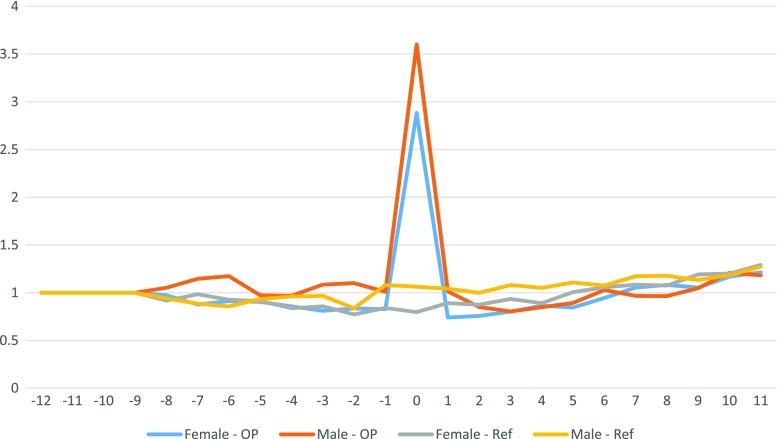
Relative change of net absenteeism in relation to the corresponding quarter in year −3

Patients with obesity had a higher absenteeism throughout the study period (Fig. 2). Both the patient cohort and the reference population displayed similar patterns of absenteeism over the 6 years studied (Fig. 3). Long-term relative differences between patients and the reference population were thus consistent with preoperative differences. Women had higher absenteeism compared to men, both in the study group and in the reference population.

### 0–90 Days Postoperatively

To capture direct effects of operation, we looked specifically at the immediate postoperative period, however including a somewhat longer time span to capture potential heterogeneity in direct complications. For the whole patient study group, an increase over mean preoperative values of 12 net days (males) or 13 net days (females) was observed for the 90 days after surgery. Further classification showed no association between preoperative comorbidity and the size of the increment; 12.9 net days of absenteeism if no preoperative comorbidity (both sexes combined) whereas the presence of comorbidity was associated with 12.6 net days (n.s.).

### 30 Days to 3 Years Postoperatively

To follow long-term consequences, the immediate postoperative period was excluded. Simple logistic regression was used to study what characterized those with more net sick leave during FU between 30 days and 3 years. Female sex (OR 1.5, CI 1.3–1.8), preoperative anti-depressant use (OR 1.5, CI 1.3–1.8), low income (OR 1.4 CI 1.2–1.8), and a preoperative history of sick leave (per day: OR 1.004, CI 1.003–1.004) were associated with more sick leave in the FU period. The preoperative presence of T2D did not predict any further increase in absenteeism (OR 1.2, CI 1.0–1.5) in the FU period.

**Fig. 4:**
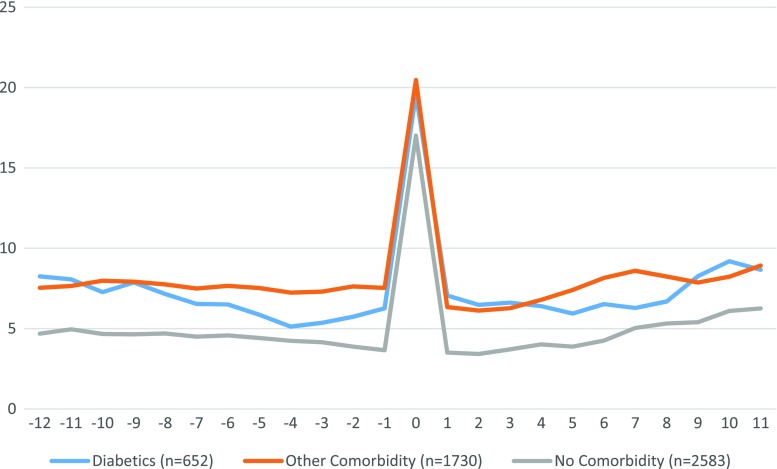
Number of net absenteeism days per 3-month period, −3 to +3 years from operation, by pre-op health status

**Fig. 5:**
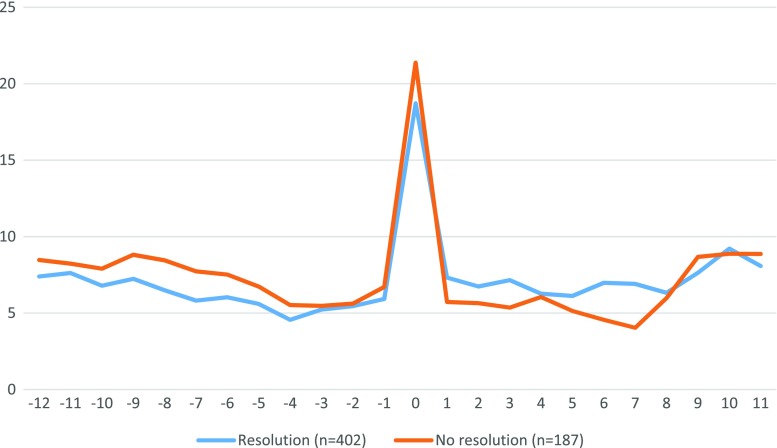
Number of absenteeism days per 3-month period, only diabetics, by post-op diabetes resolution

Patients with preoperative comorbidity had more sick days than those without, both pre- and postoperatively (Fig. 4). There were no obvious quantitative or qualitative differences between patients with diabetes and those with other types of comorbidity. Further analysis of absenteeism for patients with diabetes showed no differences in postoperative absenteeism between those with and those without diabetes resolution at the 1-year postoperative FU (Fig. 5).

## Discussion

Bariatric surgery is well known to give health benefits [1–6]. Medical care is also obviously coupled to direct as well as indirect costs, but it has been suggested that the cost per quality-adjusted life year is low in bariatric surgery [7] and that bariatric surgery may cause a long-term reduction in societal costs [7]. The operation can lead to complications but the cost of such complications is difficult to measure. Treatment of complications or re-operative surgery is often performed at other departments and may thus not be properly accounted for in any one-center surgical database. Indirect costs are even harder to quantify accurately, even though attempts have been made. A study attempting quantification of the direct costs, regardless of caregiver, has recently been launched, using other national registries in Sweden http://www.sveus.se/publikationer/rapporter/obesitaskirurgi. No study on the long-term indirect costs caused by absenteeism has been reported.

Sweden is an ideal country to study indirect costs of surgery, in this case absenteeism, since the databases for obesity surgery and sickness benefits are national and have full coverage [13, 15]. We used these databases to quantify the indirect postoperative costs of absenteeism. The study material is thus the cohort, and not a sample, of patients operated in 2010 and with access to social insurance. This year was chosen to allow for delays in 1 year FU as well as for late data entering into the systems. It was thus possible to identify all absenteeism from work leading to remuneration from the insurance system. In addition, the design of our registries enabled us to create an age-, sex-, and income-matched reference population. The operationalization means that specific medical complications were not measured, the focus being on a cohort of RYGB patients with employment, to ensure that large health problems (as well as large improvements in health) would be captured in terms of increases or decreases in work absenteeism. The rarity of incomplete cases and the use of only patients and controls with income gave us robust data. Operated patients that did not meet the inclusion criterion of employment-generated income had a higher prevalence of T2D and also a lower resolution rate. The present study has however its focus on changes on societal costs induced by bariatric surgery, and the excluded group’s lack of access to social insurance makes their possible absenteeism non-measurable and of no cost to the insurance system. Furthermore, patients unemployed before surgery but postoperatively gaining employment could not be included in the study. This seems to be less of a problem, since it has been reported that bariatric surgery causes no shifts in the rate of employment [16, 17].

Not only direct complications but also unwanted side effects to the operation could lead to increased societal costs. Any severe consequence of bariatric surgery leading to absenteeism from work is thus reflected in a database of the present design, regardless of its cause and regardless of at which level the patient is treated, inpatient, outpatient, or in primary care. For example, the recently observed complication of laparoscopic gastric bypass of internal herniation has an estimated prevalence of between 2 and 10% of patients needing a reoperation [11, 18]. In addition, Östlund et al. [10] have reported an increased incidence of hospitalization for alcohol-related disease after gastric bypass. Any such condition with its ensuing absenteeism would be captured in a study of the present design.

The SOReg database has clearly showed that most comorbidities are reduced following surgery-induced weight loss [2, 19]. The present study material shows a preoperative correlation between the existence of comorbidities and higher levels of absenteeism. Comorbidity was however not associated with absenteeism in the immediate postoperative period nor was there any tendency to indicate that the disappearance of comorbidity following surgery caused any reduction in absenteeism.

Due to the great difference in absenteeism between the study group (high absenteeism) and the reference population (low absenteeism), it can be misleading to compare changes—in absolute values—over time between the two groups. A small percentage change in absenteeism in either group would generate large changes in the absolute difference between the groups. A solution to this problem is to use the relative changes in absenteeism over time, as in the present study. The results show that the ratio of absenteeism between the patient cohort and the reference population remained constant throughout the study period. The groups followed the same pattern, albeit at different levels. Higher absenteeism among women is not unique for this patient group and is true for total sickness absenteeism in Sweden [13, 15]. Our data thus indicate that the patient group followed the national trend in sickness absenteeism both pre- and postoperatively, i.e., surgery did not alter the pattern. Our results are not quite compatible with the report by Neovius et al. [20] who found that operated patients had higher direct costs in that they consumed more hospital days the first years after surgery compared with control subjects. This difference disappeared after 7 years in a 20-year follow-up study. Their study was however based on a patient material comprising only 13% gastric bypass patients and they did not study sick leave.

*A limitation* of the present study is the lack of information on body weight in the reference population. The study was however focused on the possible differences between pre- and postoperative absenteeism, with the reference population illustrating societal changes. Another possible shortcoming is that no data were available on whether patients in the operated group had indeed been admitted to hospital or operated after the index operation, but without ensuing absenteeism. Also, short-term absenteeism, 1–2 weeks, cannot be identified using the national insurance database. The focus of the study was however societal cost for absenteeism and that could be fully accounted for, regardless of reason, so that all health consequences resulting in lost work capacity extending for more than a 2-week period were captured.

One interpretation of our results could be that RYGB can be performed in obese individuals without causing an increased long-term absence from work. The alternative interpretation is that the potential health benefits did not outweigh the postoperative complications, since RYGB did not lead to a decrease in work absenteeism. This latter is compatible with previous studies showing no shifts in employment rate after RYGB surgery [16] and no effects on the rate of disability pension [17]. This development proved however to be the case even for diabetics in our material, where patients with preoperative diabetes had an elevated absenteeism compared to patients without diabetes. After surgery, patients with diabetes resolution did not deviate towards the patient group without comorbidity, but remained in parallel to diabetic patients without resolution. This observation was unexpected and further strengthens the hypothesis that factors other than medical care are important for indirect costs to society after gastric bypass surgery. The paradox of reduced mortality after RYGB in patients with diabetes [6] and no reduction in absenteeism in the present study remains to be explained.

*In summary*, the benefits derived from bariatric surgery do not carry over to a reduction in absenteeism, but can be obtained without increasing such societal costs. Also, the postoperative absenteeism pattern of preoperative diabetic patients seems to be governed by factors other than diabetes resolution.
